# Endogenous oxytocin levels in children with autism: Associations with cortisol levels and oxytocin receptor gene methylation

**DOI:** 10.1038/s41398-023-02524-0

**Published:** 2023-06-30

**Authors:** Margaux Evenepoel, Matthijs Moerkerke, Nicky Daniels, Viktoria Chubar, Stephan Claes, Jonathan Turner, Bart Vanaudenaerde, Lynn Willems, Johan Verhaeghe, Jellina Prinsen, Jean Steyaert, Bart Boets, Kaat Alaerts

**Affiliations:** 1 KU Leuven, Department of Rehabilitation Sciences, Research Group for Neurorehabilitation, Leuven, Belgium; 2 KU Leuven, Leuven Autism Research (LAuRes), Leuven, Belgium; 3 KU Leuven, Department of Neurosciences, Center for Developmental Psychiatry, Leuven, Belgium; 4 University Psychiatric Centre, KU Leuven, Leuven, Belgium; 5 Luxembourg Institute of Health, Department of Infection and Immunity, Esch sur Alzette, Luxembourg; 6 KU Leuven, Department of Chronic Illness and Metabolism, Laboratory of Respiratory Diseases and Thoracic Surgery, Leuven, Belgium; 7 KU Leuven, Department of Development and Regeneration, Research Group Woman and Child, Leuven, Belgium

**Keywords:** Autism spectrum disorders, Diagnostic markers

## Abstract

Alterations in the brain’s oxytocinergic system have been suggested to play an important role in the pathophysiology of autism spectrum disorder (ASD), but insights from pediatric populations are sparse. Here, salivary oxytocin was examined in the morning (AM) and afternoon (PM) in school-aged children with (*n* = 80) and without (*n* = 40) ASD (boys/girls 4/1), and also characterizations of DNA methylation (DNAm) of the oxytocin receptor gene (*OXTR*) were obtained. Further, cortisol levels were assessed to examine links between the oxytocinergic system and hypothalamic-pituitary-adrenal (HPA) axis signaling. Children with ASD displayed altered (diminished) oxytocin levels in the morning, but not in the afternoon, after a mildly stress-inducing social interaction session. Notably, in the control group, higher oxytocin levels at AM were associated with lower stress-induced cortisol at PM, likely reflective of a *protective* stress-regulatory mechanism for buffering HPA stress activity. In children with ASD, on the other hand, a significant rise in oxytocin levels from the morning to the afternoon was associated with a higher stress-induced cortisol release in the afternoon, likely reflective of a more *reactive* stress regulatory release of oxytocin for reactively coping with heightened HPA activity. Regarding epigenetic modifications, no overall pattern of *OXTR* hypo- or hypermethylation was evident in ASD. In control children, a notable association between *OXTR* methylation and levels of cortisol at PM was evident, likely indicative of a compensatory downregulation of *OXTR* methylation (higher oxytocin receptor expression) in children with heightened HPA axis activity. Together, these observations bear important insights into altered oxytocinergic signaling in ASD, which may aid in establishing relevant biomarkers for diagnostic and/or treatment evaluation purposes targeting the oxytocinergic system in ASD.

## Introduction

Autism spectrum disorder (ASD) is a neurodevelopmental condition characterized by difficulties with social communication and interaction, combined with expressions of restricted and repetitive behaviors and interests. Aside from these core ASD symptoms, individuals with ASD often also display a broad range of comorbid symptoms including attachment difficulties [1] and (social) stress and anxiety [2].

While the etiology and underlying neurobiological mechanisms responsible for the heterogeneous clinical presentation of ASD are not yet fully understood, recent lines of evidence suggest that alterations in the brain’s oxytocinergic system play an important role in the pathophysiology of ASD [3]. Oxytocin is a nonapeptide produced in the paraventricular and supraoptic nuclei of the hypothalamus [4], from which oxytocinergic neurons project to various regions of the brain’s reward system and social brain circuits (e.g. amygdala) [5]. Through its widespread neuromodulatory effects, oxytocin is identified as a key mediator of affiliative and prosocial behaviors, including interpersonal bonding, attachment, and social attunement [6–8].

Initial preclinical studies have demonstrated links between aberrant oxytocinergic signaling and ASD symptom expression in rodent models of autism [9–11]. However, investigating oxytocin concentrations in biological samples (mostly plasma and saliva) collected from humans with ASD, yielded a more mixed pattern of results (see meta-analysis of Moerkerke et al. (2021) [12]). In particular, only young children with ASD (6-to-9 year old) showed reduced oxytocin levels compared to controls in four out of five studies [13–17], whereas none of the studies including 9-to-12 year old children with ASD reported a significant group difference in endogenous oxytocin levels [18–20]. Moreover, studies including adolescents (11-to-16 year old) [18–20] and adults [21, 22] with ASD revealed a mixed pattern of results, demonstrating no clear evidence for alterations in oxytocin levels in ASD.

Aside from assessments of circulating oxytocin, also variations in epigenetic modifications of the oxytocin receptor gene (*OXTR*) have been examined in relation to ASD. The functioning of the circulating oxytocin is dependent on the functioning of the oxytocin receptor [23], which itself is encoded by the *OXTR* [24]. DNA methylation (DNAm) is one of the most extensively studied epigenetic mechanisms involved in the regulation of gene transcription, such that increased DNAm frequency is generally associated with decreased gene transcription, and therefore less expression of the respective protein [25]. While evidence is sparse, a recent review on *OXTR* DNAm by Moerkerke et al. [26] pointed towards a pattern of *hyper*methylation in adults with ASD [27, 28], indicative of reduced *OXTR* expression. Contradictory, also patterns of *hypo*methylation were identified in children with ASD [29, 30], suggesting differential ASD-related alterations in *OXTR* DNAm dependent on developmental stage. However, prior studies not only varied largely in terms of population-dependent characteristics (such as age), but also with regard to the investigated DNAm *OXTR* sites (e.g. intron versus exon CpG sites) as well as other design-related factors (e.g. adopted questionnaires/scales for assessing social/ASD-related traits) [26]. However, despite the variations in investigated DNAm *OXTR* sites, previous research indicated that methylation of CpG sites -924 and -934 was highly associated with reduced *OXTR* expression, such that epigenetic variations at these sites were observed in obstructive compulsive disorder, attention deficit hyperactivity disorder and depression [31]. Also in relation to ASD, these CpG sites have been linked to reduced gene expression and patterns of hypermethylation [32].

Despite these initial insights into the role of oxytocin hormonal variations and *OXTR* epigenetic modifications in ASD, research directly investigating the link between *OXTR* DNAm and hormonal oxytocin levels remains sparse. One study by Dadds et al. (2014) demonstrated that increased *OXTR* DNAm in peripheral blood was associated with lower plasma oxytocin levels in children/adolescents with oppositional-defiant/conduct disorder (4-to-16 year old) [33]. However, another study in adults with psychotic disorders found no significant association between methylation of site -934 and plasma oxytocin [34]. Likewise, a later study with female macaques also failed to identify any significant relationship between *OXTR* DNAm of distinct CpG sites and oxytocin levels in cerebrospinal fluid [35].

Finally, several lines of evidence also suggest a significant interplay between the oxytocinergic system and signaling of the hypothalamus-pituitary-adrenal (HPA) axis in stress regulation [21]. For example, prior research in animals and humans provided indications of coupled releases of oxytocin and the HPA ‘stress-hormone’ cortisol, particularly in response to stressors, allowing oxytocin to inhibit stress-induced rises in cortisol [36]. More research is needed however, to investigate these associations further, particularly in neuropsychiatric conditions such as ASD, in which elevated cortisol levels (see [37] for recent meta-analysis) and altered oxytocinergic signaling are apparent.

To fill these gaps, the present study aimed to examine possible alterations in peripheral hormonal levels of oxytocin and cortisol (assessed from saliva samples) as well as possible alterations in *OXTR* DNAm in a representative sample of school-aged children with and without ASD. Importantly, while endocrinological levels of e.g. cortisol are known to evolve during the course of the day [38] and in response to social encounters or stressful situations [34, 39], this possibility of diurnal variations has been largely overlooked in prior research examining hormonal variations in oxytocin in ASD populations. It is however well-established that also oxytocin levels can covary depending on the impact of experimental (social) stimulations [40]. The present study will therefore assess, for the first time, the interplay between oxytocin and cortisol hormonal levels in children with ASD, at two time points, either at ‘baseline’ in the morning or in the afternoon, after a (mildly stressful) social interaction session.

## Methods

This study was approved by the Ethical Committee for Biomedical Research at the University of Leuven, KU Leuven (S61358) in accordance with The Code of Ethics of the World Medical Association (Declaration of Helsinki). Written informed consent from the parents and assent from the child were obtained prior to the study.

### Participants

Eighty children with a formal diagnosis of ASD, aged between 8–12 years, were recruited through the Leuven Autism Expertise Centre at the Leuven University Hospital between July 2019 and January 2021. Also, 40 age- and sex-matched peers without ASD (control) were recruited. The adopted sample size (80 ASD, 40 controls) will allow detecting medium-to-large diagnostic-related effects (independent samples) with alpha set at 0.05 and power at 0.8 (estimated using G*Power).

The main inclusion criteria comprised a clinical diagnosis of ASD (only for children with ASD), premenstrual girls, intelligence quotient (IQ) above 70, and native Dutch speaker. The main exclusion criteria comprised a history of any neurological disorder (stroke, concussion, epilepsy, etc.), any significant physical disorder (liver, renal, cardiac pathology), or any neuropsychiatric diagnosis (only for control children). The diagnosis of ASD was established by a multidisciplinary team (child psychiatrist and/or expert neuropediatrician, psychologist, speech/language pathologist and/or physiotherapist) based on the strict criteria of the DSM-5 (Diagnostic and Statistical Manual of Mental Disorders) [41].

In addition, the Autism Diagnostic Observation Schedule (ADOS-2) [42] was acquired (Table 1). For all children, estimates of intelligence were acquired using four subtests of the Wechsler Intelligence Scale for Children, Fifth Edition, Dutch version [43] (Table 1).

**Table 1:** Participants’ characteristics.

	ASD group		Control group		Independent t-test	
	*N*	Mean ± SD	*N*	Mean ± SD	t-value	*p*-value
**Age**	80	10.5 ± 1.3	40	10.3 ± 1.3	0.79	0.431
**IQ**						
Verbal IQ	78	107.7 ± 15.2	40	117.3 ± 12.2	−3.44	<0.001***
Performance IQ	79	102.3 ± 14.1	40	107.8 ± 12.2	−2.09	0.039*
**Gender**						
Girl	16 (20%)		8 (20%)			
Boy	64 (80%)		32 (80%)			
**Handedness**						
Left	10 (12%)		6 (15%)			
Right	70 (88%)		34 (85%)			
**ADOS-2**						
Social affect	65	7.3 ± 3.7		—		
Restricted and repetitive behavior	65	1.9 ± 1.2		—		
Total	65	9.4 ± 4.1		—		
					MWU-test	
					Z	*p*-value
**ASD symptoms and behavioral problems**						
Social responsiveness SRS-2	80	89.2 ± 21.3	40	21.9 ± 12.7	8.83	<0.001***
Repetitive/restrictive behavior RBS-R	80	27.4 ± 15.7	40	2.5 ± 4.7	8.53	<0.001***
CBCL competences	80	17.1 ± 4.5	40	22.9 ± 4.2	−5.80	<0.001***
CBCL behavioral problems	80	72.1 ± 23.9	40	21.9 ± 13.9	8.35	<0.001***
					Independent t-test	
					t-value	*p*-value
**Biological samples**						
OT levels						
AM	78	0.78 ± 0.65	39	1.07 ± 0.49	−2.68	0.009**
PM	77	0.96 ± 0.69	40	1.02 ± 0.49	−0.49	0.629
Cortisol levels						
AM	80	−0.47 ± 0.25	40	−0.43 ± 0.24	−0.82	0.415
PM	78	−0.91 ± 0.24	40	−0.95 ± 0.26	0.84	0.400
*OXTR* DNAm						
CpG site -924	77	1.62 ± 0.06	38	1.62 ± 0.05	0.31	0.758
CpG site -934	77	1.83 ± 0.05	38	1.83 ± 0.04	−0.36	0.717

Data are shown as mean ± standard deviation. *ASD* autism spectrum disorder, *IQ* Intelligence Quotient, *ADOS-2* Autism Diagnostic Observation Schedule, *SRS-2* Social Responsiveness Scale, *RBS-R* Repetitive Behavior Scale-Revised, *CBCL* Child Behavior Checklist, *AM* Anti Meridiem, *PM* Post Meridiem.

All biological samples were log-transformed.

*p < .05.

**p < .01.

***p < .001.

Children were also thoroughly characterized on autism symptom domains, using the parent-reported versions of the *Social Responsiveness Scale*, second edition (SRS-2) [44, 45] and the *Repetitive Behavior Scale-Revised* (RBS-R) [46]. Also, the parent-rated *Child Behavior Checklist* (CBCL) was obtained to assess behavioral problems and competences [47] (see Supplementary Methods for detailed descriptions of the adopted questionnaires).

As outlined in Table 1, groups were matched on age and sex, although verbal and performance IQ were overall higher in the control group. As expected, children of the ASD group demonstrated significantly higher scores on the parent-rated SRS-2 and RBS-R, indicating more social impairments and more frequent expressions of restricted and repetitive behavior (*p* < .001, Table 1). The parent-rated CBCL also showed diagnosis-related effects, indicating more severe deficits in the ASD group, compared to the control group (*p* < .05, Table 1).

Note that the (biological) data collected for the current report were part of a larger clinical study including additional neurophysiological assessments (see also next section).

### Oxytocin and cortisol salivary concentrations

For each child, salivary samples were acquired at two time points: (i) an AM sample, acquired at home, in the morning, within 30 min after awakening and before breakfast; and (ii) a PM sample, acquired in the afternoon, after completion of an experimental session performed at the Leuven University hospital. Importantly, the PM sample was collected within 30 min after finalizing an experimental test session, consisting of a semi-structured social interaction with an unknown experimenter, which could be experienced as moderately stressful, especially to the pediatric participants (see Supplementary Methods).

Salivary samples were collected using Salivette cotton swabs (Sarstedt AG & Co.). For the analysis of salivary oxytocin levels, the commercial enzyme immunoassay oxytocin ELISA kit of Enzo Life Sciences, Inc. was used, similar to prior studies [48–50]. All sample extraction and concentration procedures were conducted in accordance with the manufacturer’s instructions. Measurements were performed on undiluted samples (100 μl), and sample concentrations were calculated according to plate-specific standard curves.

Analysis of the cortisol levels was performed using the commercial enzyme immunoassay Cortisol ELISA kit of Salimetrics, Europe. Measurements were performed on undiluted samples (25 μl), and sample concentrations were calculated according to plate-specific standard curves. More detailed information regarding the salivary collection procedures and analyses is provided in Supplementary Methods.

### DNA methylation of the oxytocin receptor gene

Additional salivary samples were obtained via the Oragene DNA sample collection kit (DNA Genotek Inc., Canada) to address epigenetic variations in the level of methylation (DNAm) at two CpG sites (-934 and -924) of *OXTR* (hg19, chr3:8,810,729–8,810,845).

After data collection, 200 ng DNA was extracted from the samples, and bisulfite was converted following the manufacturer’s protocol (EZ-96 DNAm Kit, Zymo Research, Irvine, CA, USA). Bisulfite-converted DNA was stored at −80 °C until further analysis. Next, the levels of methylation at two CpG sites (i.e., -934 and -924) of *OXTR* (hg19, chr3:8,810,729–8,810,845) were determined using Pyrosequencer (Qiagen, Hilden, Germany) and analyzed using Pyromark Q96 software. Laboratory procedures and analyses were conducted in accordance with the manufacturer’s protocols and software settings (e.g. for determining unreliable samples) [47]. Protocols for the PCR amplification and Pyrosequencing analysis were adapted from Krol et al. (2019) [51]. More information regarding the adopted PCR primers can be found in Supplementary Methods.

### Data handling and statistical procedures

Prior to analysis, hormonal and DNAm data were log-transformed (log10) to deal with skewed data. Next, diagnosis-related differences were assessed by subjecting oxytocin and cortisol hormonal data to repeated-measure analyses-of-variance (ANOVA) with the between-subject factor group (ASD, control) and the within-subject factor time point (AM, PM) (see Supplementary Tables 1-2 for full ANOVA models).

*OXTR* DNAm data were subjected to independent sample t-tests to examine diagnosis-related differences in DNAm at CpG site -924 and -934.

Pearson correlation analyses were performed to assess associations between the two hormonal systems in children with or without ASD and between hormonal levels and *OXTR* DNAm. Exploratory, also correlation analyses between oxytocin hormonal levels and behavioral scales (SRS-2, RBS-R, CBCL) were examined.

Finally, to examine the potential impact of person-dependent factors, such as age, verbal and performance IQ, secondary analyses were performed, including these variables as dimensional covariates in the performed statistical analyses. Overall, for all reported analyses, the observed results patterns remained qualitatively similar with inclusion of these covariates (data not reported).

All statistical analyses were executed with SPSS (version 28.0, IBM). Due to the exploratory nature of the reported analyses, all results are reported at an uncorrected statistical threshold of *p* > .05.

## Results

### Diagnosis-related differences

#### Oxytocin levels

**Fig. 1:**
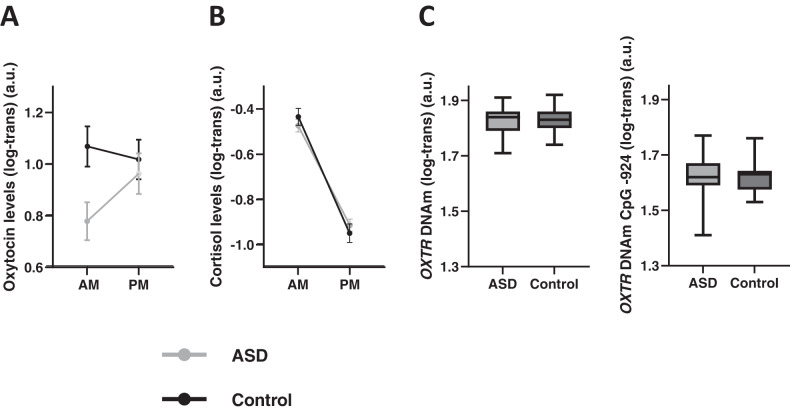
Diagnosis-related differences. Salivary oxytocin **A** and cortisol hormonal levels **B**, as well as OXTR gene methylation data **C** are visualized separately for children with and without ASD (control). Hormonal concentrations are visualized separately for the morning sample (AM), and the afternoon sample (PM), acquired after a mildly stress-inducing social interaction session. Methylation frequency of the OXTR gene is visualized separately for CpG site -934 and CpG site 924. **A** Oxytocin levels were significantly higher in the control group, compared to the ASD group, for the morning AM sample, not for the PM sample collected after the social interaction session. While oxytocin levels remained overall constant across AM and PM measurements in the control group, the ASD group displayed a steep increase in oxytocin levels from the AM measurement to the PM measurement. **B** Cortisol levels were not significantly different between the ASD and the control group. Both groups displayed a similar cortisol peak in the morning and a similar level of cortisol at the PM measurement, after finalizing the social interaction session. **C** *OXTR* DNAm at CpG sites -934 and -924 were not significantly different between the ASD group and the control group.

A repeated-measure ANOVA revealed no main effects of group (*F*(1,113) = 2.45; *p* = .120) or time-point (*F*(1,113) = 0.87; *p* = .354). A significant group x time point interaction (*F*(1,113) = 4.23; *p* = .042) was noted, indicating significantly lower morning oxytocin levels in ASD, compared to control children (AM sample, independent-samples *t*(115) = -2.68; *p* = .009, Fig. 1A and Table 1) but no differential afternoon oxytocin levels in ASD, compared to control children (PM sample *t*(115) = -0.49; *p* = .629, Fig. 1A and Table 1). Notably, while oxytocin levels remained overall constant from AM to PM in the control group (dependent-samples *t*(38) = -0.67; *p* = .507), the ASD group displayed a steep and significant increase in oxytocin levels from AM to PM (*t*(75) = 2.61; *p* =.011) (Fig. 1A).

#### Cortisol levels

A repeated-measure ANOVA revealed no significant main effect of group (*F*(1,116) = 0.002; *p* = .965) nor group x time point interaction (*F*(1,116) = 1.86; *p* = .175) (Fig. 1B and Table 1). A significant main effect of time point was evident however (*F*(1,116) = 286.83; *p* < .001), indicating that in both groups, a steep decline in cortisol levels was evident from AM to PM. Accordingly, it appears that - as a group - children with or without ASD display a similar cortisol peak in the morning and a similar pattern of cortisol diminishment during the course of the day.

#### *OXTR* DNAm

No significant diagnosis-related differences in *OXTR* DNAm were evident between the ASD and control group, either at CpG site -934 (*t*(113) = −0.33; *p* = .745) or at CpG site -924 (*t*(113) = 0.44; *p* = .659) (Fig. 1C).

### Association between oxytocin and cortisol hormonal levels

**Fig. 2:**
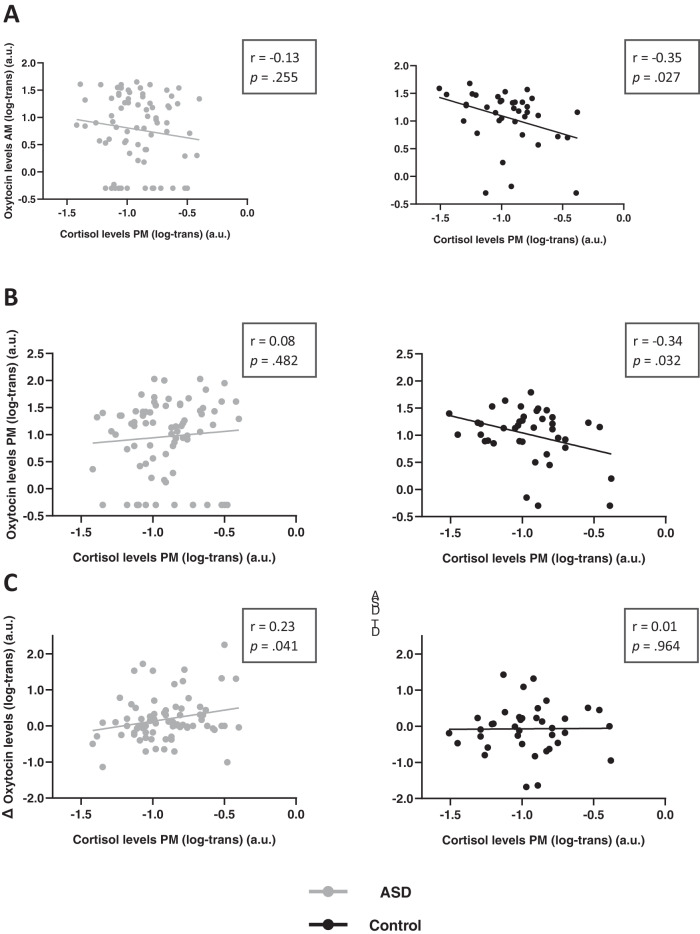
Association between oxytocin and cortisol hormonal levels. Relationships between oxytocin and cortisol hormonal levels are visualized separately for children with ASD and typically developing children (control). **A** In the control group, oxytocin levels in AM were significantly associated with cortisol levels at PM, indicating that high morning oxytocin was predictive of low ‘stress-induced’ PM cortisol levels. Relationships were only significantly evident in the control group, not in the ASD group. **B** Oxytocin levels at PM were also significantly associated with cortisol levels at PM, indicating that higher afternoon oxytocin levels were associated with lower ‘stress-induced’ cortisol levels. Also here, the relationship was only evident in the control group, not in the ASD group. **C** Finally, in the ASD group, the observed marked increases in oxytocin levels during the day (from the AM to the PM assessment) were significantly associated with higher afternoon ‘stress-induced’ cortisol levels.

In the control group, a tight interaction between oxytocin and cortisol hormonal systems was evident, indicating that high morning oxytocin levels (AM sample) were predictive of lower ‘stress-induced’ cortisol levels (PM sample) (Pearson *r* = −0.35; *p* = .027) (Fig. 2A). Higher oxytocin levels in the afternoon, i.e. after the (mildly stressful) social interaction session, were also significantly associated with reduced cortisol levels in the afternoon (PM sample) (*r* = −0.34; *p* = .032) (Fig. 2B).

Strikingly, these associations were only evident in the control group, not in the ASD group, suggesting a lack of coupling of these hormonal systems in children with ASD (all, *r* < 0.13; *p* > .05). Supplementary Table 3 reports a full overview of the assessed associations between oxytocin and cortisol levels, separately for each group, including a direct comparison of correlation values between ASD and control groups.

Notably, considering the marked AM to PM increase in oxytocin levels seen in the ASD group, we additionally explored whether these AM to PM changes in oxytocin levels were potentially related to AM to MP changes in cortisol levels and/or overall cortisol levels.

While AM to PM changes in oxytocin were not directly related to AM to PM changes in cortisol (*r* < 0.13; *p* > .05), it was revealed that only in the ASD group (*r* = 0.23; *p* = .041), not in the control group (*r* = 0.01; *p* = .964), the AM to PM rise in oxytocin levels during the day was associated to higher overall cortisol levels at PM (Fig. 2C).

### Associations between hormonal levels and OXTR DNAm

#### CpG site -924

**Fig. 3:**
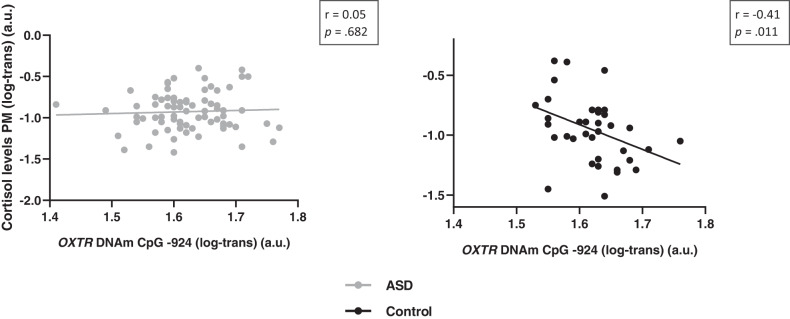
Associations between hormonal levels and *OXTR* DNAm. Associations between hormonal levels and methylation of the *OXTR*, visualized separately for children with and without ASD. In the control group, not in the ASD group, a significant negative association was evident between *OXTR* methylation of CpG site -924 and cortisol levels, indicating that children with lower methylation frequency (higher *OXTR* expression) displayed higher afternoon cortisol levels.

Only in the control group (*r* = −0.41; *p* = .011), not in the ASD group (*r* = 0.05; *p* = .682), a significant association between *OXTR* DNAm of site -924 and PM cortisol levels was evident, indicating that children with lower methylation frequency of CpG site -924 (higher *OXTR* expression) displayed higher afternoon cortisol levels (Fig. 3). *OXTR* DNAm of site -924 was not significantly associated with AM cortisol levels or AM/PM oxytocin levels either in children with or without ASD (all, *r* < 0.07; *p* > .05; see Supplementary Table 3 for a full overview).

#### CpG site -934

Methylation of CpG site -934 was not significantly associated with AM or PM oxytocin or cortisol levels, either in children with or without ASD (all, *r* < 0.08; *p* > .05, see Supplementary Table 3).

### Association between oxytocin levels and behavioral data

Despite diagnosis-related group differences in oxytocin hormonal levels, correlation analysis did not reveal dimensional relationships between ASD symptom severity (SRS-2 or RBS-R) and morning or afternoon oxytocin levels, either in the ASD or control groups (all *r* < 0.26; *p* > .05). However, looking at dimensional variations in behavioral problems and competences (CBCL), a significant association was evident between CBCL competences and oxytocin levels in the afternoon in the control group (*r* = 0.469; *p* = .002), but not in the ASD group (*r* = 0.130; *p* = .261), indicating that higher levels of oxytocin in the afternoon are related to higher social competences and competences at school.

When performing subgroup analyses, including only a subsample of boys with and without ASD, the diagnosis-related differences and association analyses yielded a qualitatively similar pattern of results (see Supplementary Table 4).

## Discussion

In the current study, we investigated alterations in oxytocin and cortisol hormonal systems as well as *OXTR* DNAm in school-aged children with ASD, compared to a group of age and sex-matched control children.

Only in terms of ‘baseline’ oxytocin, children with ASD displayed reduced levels of peripheral oxytocin assessed in the morning, not in terms of afternoon oxytocin levels, assessed after induction of a mildly stressful social interaction session. These findings therefore provide indications that only *‘trait-dependent’* oxytocin levels, but not stress-reactive *‘state-dependent’* levels of oxytocin are altered in ASD. Further, while overall cortisol levels were similar in children with and without ASD, differential couplings between the oxytocin and cortisol hormonal system were evident, indicating that only in the control group, not in the ASD group, higher oxytocin levels were predictive of dampened afternoon ‘stress-induced’ cortisol levels. Finally, DNAm at two distinct CpG sites of *OXTR* were not altered in children with ASD, although note that only in the control group, methylation frequency was significantly associated with hormonal reactivity of the cortisol system.

Diagnosis-related differences in oxytocin hormonal levels were evident in children with ASD, albeit exclusively in terms of ‘*trait-dependent*’ morning oxytocin, not in terms of ‘*state-dependent*’ afternoon oxytocin levels, measured after an experimental social interaction session. Prior meta-analytic analyses examining altered oxytocin levels in ASD yielded a mixed pattern of results, predominantly depending on the age of the included participants [12]. While it is difficult to directly compare studies, the current data provide important indications that also the time of the day, as well as the particular experimental and social context (with or without experimental manipulation), are important factors to consider for explaining possible variations in oxytocin levels.

A closer inspection of the recent meta-analysis of Moerkerke et al. (2021) [12] showed that only in 11 out of 18 studies the timepoint or time range of sampling was reported. Of these studies, six reported to have acquired ‘morning’ oxytocin samples (AM) [13, 15, 17, 20, 21, 52], and four of these six reported similar group-related differences as in our study, indicating lower morning oxytocin levels in ASD compared to the control group [13, 15, 21, 52]. In accordance with our study, the only two studies that indicated to have sampled oxytocin in the afternoon (PM), reported no significant group differences [18, 19]. Further, in the remaining studies that reported sampling timings, it appeared that acquisition timing was not standardized across individuals (i.e., ranging from morning to afternoon), therefore rendering direct comparisons with our current results was difficult [22, 53, 54]. As the time point and context of sampling may have an important impact on observed levels of oxytocin and correlations with other outcome measures, future studies are urged to report this important design-specific information in more detail.

With regard to cortisol levels, in the current study, no differences in morning or afternoon cortisol levels were identified in children with or without ASD, indicating no overall alterations in HPA axis reactivity, as demonstrated before ([21], although see [55]). Notably, depending on diagnosis, differential couplings between the oxytocinergic system and the HPA axis (as indexed with cortisol) were evident, indicating that only in the control children, not in children with ASD, heightened (morning) oxytocin levels were predictive of dampened ‘*state-dependent*’ release of cortisol. Particularly, this negative coupling was strongest between morning oxytocin and afternoon cortisol levels measured after the mildly stress-inducing social interaction session. These results therefore suggest that higher ‘*trait-dependent*’ levels of morning oxytocin, seen in control children, may constitute an important *protective* mechanism to buffer HPA axis activity upon (social) stressors encountered during the day. In children with ASD, on the other hand, this protective oxytocinergic HPA-stress attenuation mechanism may be dysfunctional or absent, considering the overall diminished levels of morning oxytocin in the children with ASD, and the apparent lack of coupling between oxytocin and cortisol systems in this group.

Overall, our observations of oxytocinergic dampening of HPA-cortisol responses are in line with prior studies showing acute, dampening effects of intranasal oxytocin administration on cortisol reactivity to laboratory tasks [56, 57]. Interestingly, meta-analytic evidence showed that the attenuating effects were most pronounced in response to challenging laboratory tasks that produced a robust stimulation of the HPA axis, as well as in clinical populations with ASD or schizophrenia, compared to neurotypicals [56]. This latter observation is important, as the authors interpreted this finding to reflect an increased sensitivity to the exogenously administered oxytocin among those with a clinical diagnosis, presumably because they may present lower baseline oxytocin levels. Albeit indirectly, our current observations corroborate this interpretation by showing that morning levels of salivary oxytocin are indeed significantly diminished in children with ASD. Exogenously administered oxytocin may therefore be particularly effective in ASD, i.e., for restoring the otherwise dysfunctional oxytocinergic HPA stress attenuation.

Notably, while diminished levels of oxytocin were initially noted in children with ASD, a significant increase was observed from AM to PM, reaching a similar level of concentration as noted in control children. In control children, levels remained overall stable throughout the day. While speculative, the steep increase in oxytocin release in ‘*state-dependent*’ afternoon oxytocin, compared to ‘*trait-dependent*’ morning oxytocin, can be hypothesized to reflect a compensatory mechanism of *reactive* oxytocin release upon the mildly stressful social interaction session. Indeed, while absolute oxytocin and cortisol levels were not significantly associated in children with ASD, exploratory analyses confirmed that, in the ASD group, higher state-dependent, afternoon cortisol levels were paralleled by a steeper morning to afternoon rise in oxytocin levels, likely reflective of a coupled co-release of cortisol and oxytocin upon stress-inducing tasks, as demonstrated before in both animals [58] and humans [36]. In this view, the parallel release of oxytocin upon HPA activation may be considered a *reactive* stress-regulatory mechanism, which—over time—may facilitate a dampening and further reduction of the cortisol-induced stress response. As highlighted by Brown et al., positive associations between oxytocin and cortisol levels upon acute stress induction are thought to be reflective of an initial co-release, for subsequently dampening stress responses and facilitating coping behaviors [36]. These and our findings, therefore, highlight the importance of studying the interaction among oxytocinergic and HPA stress systems from a dynamic, time-varying perspective, rather than studying static levels of a single marker.

To sum up, in children with ASD, the *positive* association between the steep rise in oxytocin levels and stress-induced HPA-cortisol levels may be indicative of a *reactive* stress-regulatory mechanism. In control children, on the other hand, the identified *negative* association between trait (morning) levels of oxytocin and stress-induced HPA-cortisol levels are likely reflective of a *protective* oxytocinergic stress-regulatory mechanism.

Aside from hormonal characterizations, also variations in DNAm frequencies of distinct CpG sites of *OXTR* were assessed, but no significant group differences were identified. These results according to observations of a recent study by Siu et al. (2021), neither showing significant group differences in the level of methylation of CpG sites -934 and -924 in children and adolescents with and without ASD [30]. Despite some variation, a recent review pointed towards differential patterns of hypo- versus hyper-methylation, respectively in children and adults with ASD, although the number of included studies was limited, and overall sample sizes were small to modest [26]. Also, considerable design-related variations were noted by the authors, e.g. in terms of included CpG sites, rendering it difficult to draw conclusive interpretations regarding ASD diagnosis-related alterations in *OXTR* DNAm based on the existing evidence.

In terms of relationships between hormonal levels and *OXTR* DNAm, only the control group displayed a significant negative association between *OXTR* DNAm (at CpG site -924) and state-dependent (afternoon) cortisol levels, indicating that lower methylation frequencies were associated with higher cortisol levels. This finding accords with a prior study identifying a similar negative relationship between stress-induced cortisol and *OXTR* DNAm frequency in individuals with social anxiety disorder (SAD) [59]. In the latter study, the pattern of *OXTR* hypomethylation associated with SAD was interpreted to be reflective of a compensatory mechanism, i.e., facilitating an upregulation of oxytocin receptors to cope with heightened HPA axis activity in SAD. More research is needed, however, to corroborate this interpretation and pattern of results in future studies.

While the current study provides important new insights into the oxytocinergic system of children with ASD and its interactions with HPA stress reactivity, the following limitations and recommendations are noted. First, as indicated, the included children reflected a rather homogenous group of high-functioning children (IQ > 70) within a tight pre-pubertal age range, rendering generalizability of the identified effects to other age ranges or to populations of children with ASD and co-occurring intellectual disability uncertain. Examining the observed effects, also in children with co-occurring intellectual disability would be of high relevance however, considering the high prevalence of this co-occurring condition in the autistic population, i.e. estimated that 70% of people with ASD would also have intellectual disabilities [60]. Further, while the included number of boys and girls in our sample reflected the well-documented four-to-one male bias in ASD prevalence [61], future studies should more closely examine the impact of biological sex on the observed effects. While subgroup analysis of the current cohort, including only boys with ASD, revealed a qualitatively similar pattern of diagnosis-related effects (see Supplementary Table 4), future research with larger samples of girls with ASD is needed, especially given prior reports of sex-related differences in oxytocinergic function [62].

While the inclusion of multiple salivary sampling time points (both in the morning and afternoon) was considered a strong asset for examining dynamic trait (morning) versus state-dependent hormonal levels, it is noted that any future design would benefit from amplifying the sampling frequency even further, allowing an even more fine-grained sampling of dynamic changes in hormonal systems interactions [36]. Indeed, it is noted that the morning and afternoon sampling may have shown some inherent differences, e.g. the morning sampling was always performed on an empty stomach, immediately after awakening, whereas the PM samples were recorded after the experimental session upon a more variable feeding status. While these differences are anticipated to be reflective of real-life variations, it should be interesting for future studies to examine the effect of circadian rhythms on variations in hormonal levels more closely, e.g. by controlling more explicitly for the feeding status and activity levels of the children.

To conclude, this study provides new insights into altered oxytocinergic signaling in children with ASD and its interactions with the cortisol HPA stress system. Results provide important indications that only ‘*trait-dependent*’ oxytocin levels, but not (social) stress-induced ‘*state-dependent*’ levels of oxytocin are altered in ASD. These observations suggest that control children without ASD may rely more on a *protective* stress-regulatory mechanism, involving high circulating trait levels of oxytocin for buffering stress reactivity, whereas children with ASD may rely more heavily on a *reactive* stress regulatory mechanism, involving more pronounced stress-induced, state-dependent releases of oxytocin upon HPA-cortisol activation, i.e., for reactively dampening stress responses and facilitating coping behaviors. While more work is needed, the identified results may aid in establishing relevant biomarkers for diagnostic and/or treatment evaluation purposes, as well as for the development of new (personalized) treatment approaches for targeting the oxytocinergic system in ASD.

Preprint was already available before the publication of this manuscript at medRxiv [63].

## Supplementary information

Supplementary material
